# Impacts of Copper Deficiency on Oxidative Stress and Immune Function in Mouse Spleen

**DOI:** 10.3390/nu17010117

**Published:** 2024-12-30

**Authors:** Xiaocong Li, Xin Zeng, Wanqin Yang, Peng Ren, Hengxiao Zhai, Heng Yin

**Affiliations:** 1 School of Life Science and Engineering, Southwest University of Science and Technology, Mianyang 621010, China; xclllyl19@gmail.com (X.L.);; 2 Tianfu Institute of Research and Innovation, Southwest University of Science and Technology, Chengdu 610299, China

**Keywords:** copper deficiency, mouse, spleen, oxidative stress

## Abstract

Introduction: Copper is an essential trace element crucial for enzyme synthesis and metabolism. Adequate copper levels are beneficial for maintaining the normal immune function of the spleen. Copper deficiency disrupts the metabolic processes within the spleen and impairs its immune function. This research examines the impact of copper deficiency on the spleen and the potential recovery following copper supplementation. Methods: Weaned mice underwent a 4-week copper-deficient diet, succeeded by 1-week of copper repletion via intraperitoneal copper sulfate injection. Histological examination was used to assess pathological changes in the spleen. Biochemical assays were performed to measure oxidative stress levels in the spleen. ELISA, qPCR, and Western blot were employed to examine alterations in inflammatory markers, immune indicators, and oxidative regulatory factors across various levels. Results: Copper deficiency caused histological damage to the spleen, altered the expression of oxidative stress regulatory pathways (Nrf2, Keap1, and HO-1), and affected the expression of key inflammatory enzymes (iNOS, COX2) and transcription factor NF-κB, leading to oxidative damage. This was reflected by decreased levels of SOD, GSH, and T-AOC, along with increased levels of CAT and MDA. The levels of inflammatory cytokines IL-1β, TNF-α, IL-6, and IL-8 were notably increased. Copper supplementation significantly improved these changes. Conclusions: Copper deficiency leads to spleen tissue damage in mice, affecting the Nrf2 regulatory pathway and inducing oxidative damage. Subsequent copper supplementation with copper sulfate effectively ameliorates the damage caused by copper deficiency.

## 1. Introduction

Trace elements, like copper, zinc, iron, and phosphorus, are crucial for growth, development, and health maintenance in animals and humans, significantly contributing to various physiological processes [1,2]. Copper (Cu) is the third most abundant trace element in animals and humans, essential for the proper functioning of various organs [3]. As a vital trace element, it is widely involved in redox reactions, cellular energy metabolism, neurotransmission, immune regulation, and other vital biological processes. Under normal conditions, copper digestion, absorption, and metabolism in the body are tightly regulated, ensuring relatively stable copper levels [4].

However, disruptions in copper homeostasis can occur due to genetic, disease, or environmental factors. Copper overload can lead to severe consequences. For instance, in Wilson’s disease, copper accumulates in multiple organs, and prolonged copper accumulation can cause organ damage or even carcinogenesis [5,6,7]. Additionally, excessive copper accumulation has been observed in Alzheimer’s disease patients, with dysregulated copper homeostasis potentially contributing to its pathogenesis [6]. In cancer, elevated copper levels may promote tumor growth and metastasis [8]. In contrast to copper overload, copper deficiency caused by a monotonous diet, absorption disorders in humans, or improper feeding in animals can also lead to dysfunction in multiple systems, resulting in conditions such as anemia [9]. Recent research indicates that copper interacts with the cytoplasmic pattern recognition receptor alpha-kinase 1 in host cells, playing a role in mediating innate immune functions [10]. Consequently, a deficiency in copper could hinder this process. In addition, Cu deficiency may lead to a reduction in IL-2, affecting T cell proliferation, which in turn impacts humoral immune function [11]. Maintaining appropriate copper levels is thus critical for preserving normal immune function.

Oxidative stress occurs when reactive oxygen species (ROS) and free radicals exceed the antioxidant defense system’s capacity, resulting in cellular and tissue damage [12]. Copper functions as a structural and catalytic cofactor for enzymes like superoxide dismutase (SOD), tyrosinase, and cytochrome c oxidase, and plays a role in synthesizing heme, collagen, and neurotransmitters. Copper also regulates the metabolism of other trace elements and modulates immune cell activity. Excessive copper can lead to ROS production [13]. While ROS generation is a normal part of immune responses, contributing to leukocyte activation, proliferation, and cytokine secretion, excessive ROS can disrupt immune cell function, triggering immune tolerance or overactivation of the immune system, which may result in autoimmune diseases or chronic inflammation [14]. Studies indicate that copper deficiency in mice diminishes hepatic SOD and total antioxidant capacity, impairing free radical scavenging and possibly leading to mitochondrial dysfunction in cardiomyocytes [14]. Copper deficiency may induce oxidative stress and inflammation in immune organs by activating the NF-κB pathway [15].

The spleen, a critical immune organ, is involved in the development, response, and regulation of immune cells [16]. Studies have shown that excessive copper induces spleen toxicity, leading to functional impairment [17,18,19]. The impact of copper deficiency on spleen function is not well-studied. This study investigates the impact of a copper-deficient diet on the spleen in mice. The impact of subsequent copper supplementation will be assessed to elucidate the mechanisms of copper’s role in spleen function, forming a foundation for developing preventive and therapeutic strategies.

## 2. Materials and Methods

### 2.1. Animals Housing

The Animal Protection and Ethics Committee of the Southwest University of Science and Technology approved all experimental procedures (approval code L2024014, 12 March 2024). Following the approval of the experimental protocol, 36 healthy, three-week-old male ICR mice (weighing 18–22 g) were obtained from Chengdu Dashuo Experimental Animal Co., Ltd, Chengdu, China. After a one-week acclimatization, mice were randomly divided into three groups: control, copper-deficient (CuD), and copper-deficient with cupric sulfate supplementation (CuD + CuSO_4_), with 12 mice per group housed in centralized cages, and 4 mice per cage. The period of the experiment was 5 weeks. During the experiment, the control group was continuously fed a normal diet, while the CuD and CuD + CuSO_4_ groups were fed a copper-deficient diet. The copper-deficient diet was prepared following established methods [14,20] and verified to comply with the AIN-93M nutritional standards [21], featuring a significantly lower copper content compared to the control diet. The copper levels in the peripheral blood of the mice were measured in the study by Pan et al. [20], showing a significant reduction in blood copper levels in the CuD group, confirming that the copper-deficient diet was effective. This protocol was used to ensure the adequacy of copper deficiency and supplementation in the current study. In the final week, the CuD + CuSO_4_ group received intraperitoneal injections of copper sulfate solution (10 μg/g), while the control and CuD groups received an equal volume of normal saline. The administration had no impact on the copper-deficient model, eliminating potential confounding factors in the experimental outcomes. Both the normal diet and copper-deficient diet were provided by SiPeiFu Biotechnology Co., Ltd., Beijing, China. The experiment was conducted at an ambient temperature of 22 ± 2 °C and a relative humidity of 60 ± 10%. The mice were provided access to food and water ad libitum.

### 2.2. Histological Observation

On the last day of the experiment, six mice were randomly chosen from each group, weighed, and then anesthetized with ether. Blood was collected from the eyeball, placed into a 1.5 mL clean centrifuge tube, and allowed to stand. Following blood collection, the mice were euthanized via cervical dislocation, and their spleens were excised. The connective tissue on the spleens was carefully dissected, and the spleen was weighed with a 1/10,000th scale (BSA224S, Sartorius, Göttingen, Germany). The spleen index was determined by the ratio of spleen weight (mg) to body weight (g). After weighing, the spleens were fixed in 4% paraformaldehyde. Following adequate fixation, the spleens underwent dehydration using a graded ethanol series (75%, 85%, 95%, and 100%) before being embedded in paraffin. Sections, 5 μm thick, were stained with hematoxylin and eosin (H&E). Microscopic examination assessed splenic histological alterations, with the degree of histopathological changes quantitatively scored following Elmore’s guidelines [22].

### 2.3. Peripheral Blood Inflammatory Response Factor Detection

Following adequate standing time, the serum was isolated by centrifuging the blood previously described at 3000 rpm for 10 min using a refrigerated centrifuge (5804R, Eppendorf, Hamburg, Germany). The serum levels of inflammatory factors IL-1β (BSEM-015-96T), IL-6 (BSEM-006), IL-8 (BSEM-008), and TNF-α (BSEM-004) were measured using ELISA kits purchased from Labgic Technology Co., Ltd., Beijing, China.

### 2.4. Biochemical Assays

At the conclusion of the experiment, six mice from each group were randomly chosen, and their spleens were collected and stored in liquid nitrogen. The frozen spleens were ground into a powder using liquid nitrogen, and a portion of the tissue powder was reserved for real-time quantitative PCR and Western blot, while the remaining tissue powder was used to prepare a tissue homogenate. The supernatant was collected following centrifugation of the homogenate at 3000 rpm for 10 min. Biochemical assay kits from Nanjing Jiancheng Bioengineering Institute, Nanjing, China, were utilized to assess malondialdehyde (MDA), glutathione (GSH), catalase (CAT), superoxide dismutase (SOD), and total antioxidant capacity (T-AOC) levels. The assay kit product numbers are A003–1, A006-2-1, A007-1-1, A001-2-2, and A015-2-1, respectively.

### 2.5. Real-Time Quantitative PCR Analysis

RNA was extracted from spleen tissue powder using the RNAiso Plus reagent kit (9108/9109, Takara, Otsu, Japan), according to the manufacturer’s instructions. Complementary DNA (cDNA) was synthesized with the PrimeScript™ RT reagent kit (RR047A, Takara, Japan). The SYBR Premix Ex Taq™ II reagent kit (DRR820A, Takara, Japan) was employed for DNA staining. Real-time quantitative PCR (qPCR) was performed using a CFX96 Touch Real-Time PCR Detection System (Bio-Rad, Hercules, CA, USA). Gene expression was normalized to β-actin, and relative mRNA levels were determined using the 2^−ΔΔCt^ method. The specific primer sequences used for qPCR are listed in Table 1.

### 2.6. Western Blot Analysis

An appropriate amount of spleen tissue powder was used to extract proteins with RIPA lysis buffer. Protein concentration was determined using the BCA assay kit (P0010S, Beyotime Technology, Shanghai, China) to ensure equal loading, allowing for consistent total protein amounts in each lane. Proteins were isolated using SDS-PAGE and subsequently transferred onto a nitrocellulose membrane. Following a 1 h room temperature block with 5% non-fat dry milk, the membrane was incubated overnight at 4 °C with primary antibodies targeting Nrf2 (ab137550, Abcam, Cambridge, UK), Keap1 (ab119403, Abcam), HO-1 (ab68477, Abcam), and various CST antibodies: pNF-κB (#3033), NF-κB (#8242), iNOS (#2982), COX2 (#4842), IL-1β (#12624), IL-6 (#12912), TNF-α (#3707), and β-actin (#4970), each at a 1:1000 dilution. Protein bands were visualized on a Chemidoc XRS system (Bio-Rad, Hercules, CA, USA) following a 1 h incubation with horseradish peroxidase-conjugated secondary antibody and detection using the ECL reagent kit (P0018A, Beyotime Technology, Shanghai, China). Quantitative analysis was performed, with protein expression levels normalized to β-actin expression.

### 2.7. Data Analysis

Data are expressed as mean ± standard deviation. Data analysis was conducted using one-way ANOVA with a Bonferroni post hoc test in GraphPad Prism 9.0, and corresponding images were produced. A *p*-value below 0.05 was deemed statistically significant.

## 3. Results

**Figure 1:**
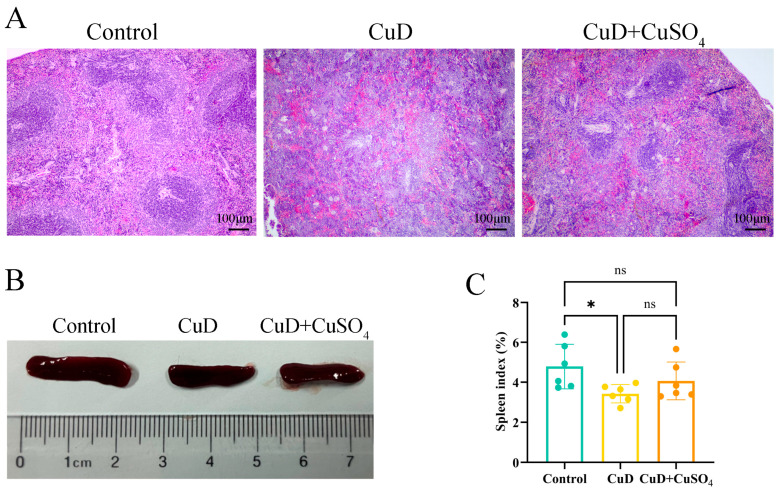
Effects of Cu deficiency and supplementation on spleen index and histological morphology in mice. (**A**) Spleen microscopic structure of mice, H&E, 100×. (**B**) Appearance and size of spleen. (**C**) Spleen index of the groups of mice. * *p* < 0.05, ^ns^ *p* > 0.05.

The histological structure, appearance, and spleen index of the mouse spleen are shown in Figure 1. Panel A shows that the spleen morphology of the control group mice is normal. The copper-deficient group (CuD) shows diminished white pulp with indistinct boundaries compared to the control group. Table 2 quantitatively scores spleen damage, showing a higher damage score in the CuD group compared to the control group. The copper sulfate supplementation group (CuD + CuSO_4_) shows some pathological changes relative to the control group, but these are less severe compared to the CuD group. The CuD + CuSO_4_ group exhibited a lower pathological score compared to the CuD group, suggesting less severe damage. Panel B shows a reduction in spleen volume in both the CuD and CuD + CuSO_4_ groups compared to the control group. Panel C presents the spleen index of the three groups. The spleen indices in both the CuD and CuD + CuSO_4_ groups are significantly lower than those in the control group, with no notable difference between the two groups.

**Figure 2:**
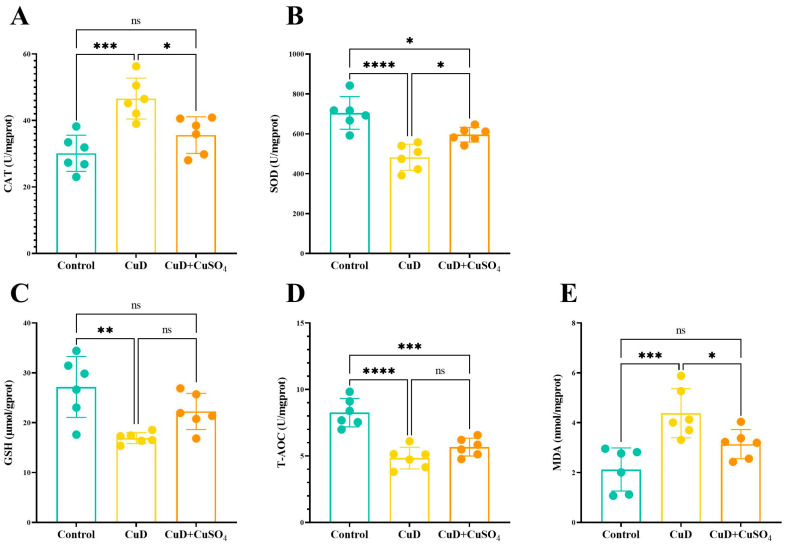
Effects of Cu deficiency and supplementation on oxidative damage of spleen in mice. (**A**–**E**) represent the CAT activity, SOD activity, GSH concentration, total antioxidant capacity, and MDA content in the spleens of mice from each group, respectively. * *p* < 0.05, ** *p* < 0.01, *** *p* < 0.001, **** *p* < 0.0001, ^ns^ *p* > 0.05.

Figure 2 illustrates the impact of copper deficiency and copper sulfate supplementation on oxidative damage markers in mouse spleens. Panel A indicates significantly elevated CAT activity in the CuD group compared to the control group. The CuD + CuSO_4_ group exhibits significantly lower CAT activity than the CuD group, and no significant difference from the control group. Panel B indicates that SOD activity is significantly reduced in both the CuD and CuD + CuSO_4_ groups compared to the control group, while the CuD + CuSO_4_ group demonstrates significantly higher SOD activity than the CuD group. Panel C indicates that GSH levels are significantly reduced in the CuD group compared to the control group, whereas the GSH level in the CuD + CuSO_4_ group showed no significant difference from either the control group or the CuD group. Panel D shows that the T-AOC levels in both the CuD and CuD + CuSO_4_ groups are significantly lower than those in the control group, with no significant difference between the two experimental groups. Panel E indicates significantly elevated MDA levels in the CuD group compared to both the control and CuD + CuSO_4_ groups, while no significant difference is observed between the control and CuD + CuSO_4_ groups.

**Figure 3:**
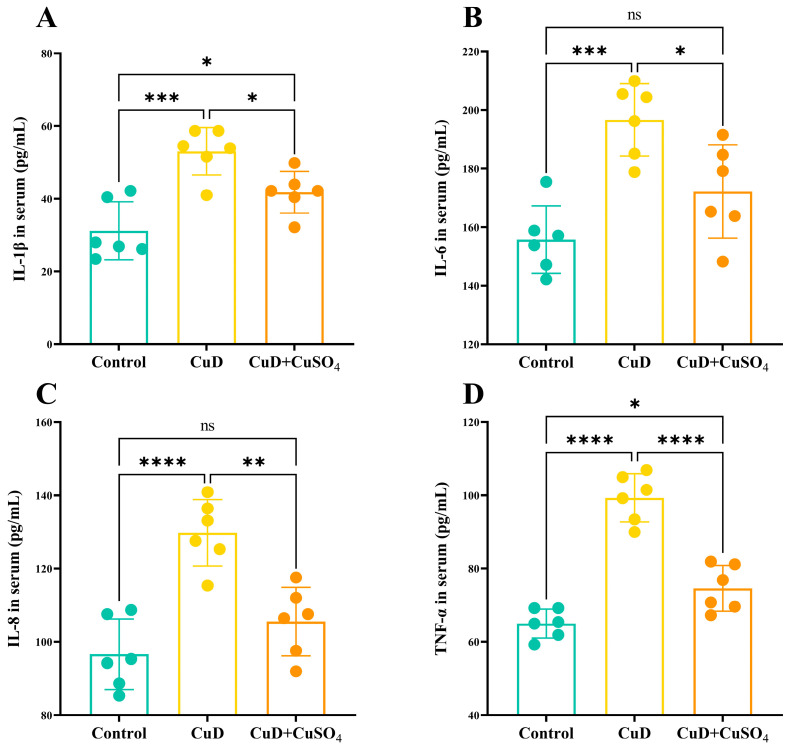
Effects of Cu deficiency and supplementation on cytokine of serum in mice. (**A**–**D**) represent concentrations of IL-1β, IL-6, IL-8, and TNF-α in mouse serum, respectively. * *p* < 0.05, ** *p* < 0.01, *** *p* < 0.001, **** *p* < 0.0001, ^ns^ *p* > 0.05.

Figure 3 presents the effect of copper deficiency and copper sulfate supplementation on peripheral blood cytokine levels in mice. Panels A and D indicate that IL-1β and TNF-α levels are significantly elevated in the CuD and CuD + CuSO_4_ groups compared to the control group. IL-1β and TNF-α levels are notably reduced in the CuD + CuSO_4_ group relative to the CuD group. Panels B and C reveal that IL-6 and IL-8 levels are significantly elevated in the CuD group compared to the control group. In contrast, the CuD + CuSO_4_ group exhibits IL-6 and IL-8 levels that are not significantly different from the control group, yet are significantly lower than those in the CuD group.

**Figure 4:**
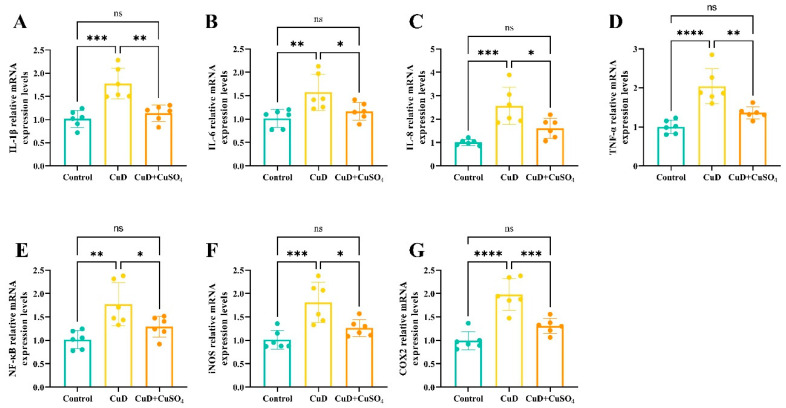
Effects of Cu deficiency and supplementation on mRNA expression of spleen inflammation-related genes. (**A**–**G**) represent the mRNA relative expression levels of IL-1β, IL-6, IL-8, TNF-α, NF-κB, iNOS, and COX2 in the spleens of mice, respectively. * *p* < 0.05, ** *p* < 0.01, *** *p* < 0.001, **** *p* < 0.0001, ^ns^ *p* > 0.05.

Figure 4 displays the changes in relative expression levels of cytokines in mouse spleen as detected by quantitative PCR. The CuD group exhibits a significant increase in the relative expression of IL-8, IL-1β, TNF-α, IL-6, NF-κB, iNOS, and COX2 compared to the control group. In the CuD + CuSO_4_ group, the relative expression of the seven cytokines is significantly reduced compared to the CuD group, with no notable differences observed between the CuD + CuSO_4_ group and the control group.

**Figure 5:**
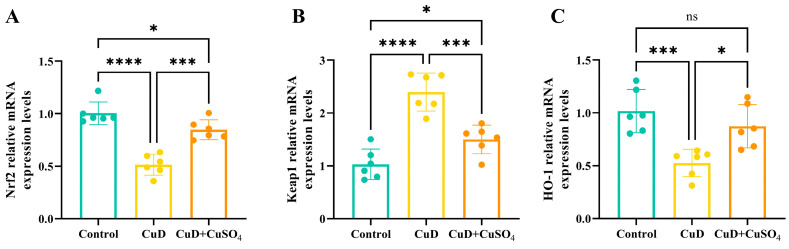
Effects of Cu deficiency and supplementation on mRNA expression of spleen nrf2 signaling pathway. (**A**–**C**) represent the mRNA relative expression levels of Nrf2, Keap1, and HO-1 in the spleens of mice, respectively. * *p* < 0.05, *** *p* < 0.001, **** *p* < 0.0001, ^ns^ *p* > 0.05.

Figure 5 indicates that the CuD group exhibits significantly reduced mRNA expression levels of Nrf2 and HO-1, whereas Keap1 expression is significantly elevated compared to the control group. In the CuD + CuSO_4_ group, mRNA expression levels of Nrf2 and HO-1 are significantly elevated, whereas Keap1 expression is notably reduced compared to the CuD group. In the CuD + CuSO_4_ group, Nrf2 mRNA expression is significantly reduced, Keap1 expression is significantly elevated, and HO-1 expression remains unchanged compared to the control group.

**Figure 6:**
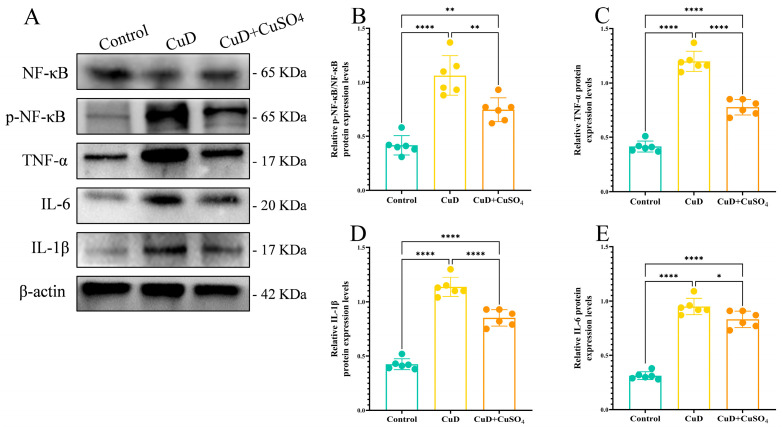
Impact of Cu deficiency and supplementation on spleen inflammation-related protein expression. (**A**) Protein levels of p-NF-κB, NF-κB, TNF-α, IL-1β, and IL-6 in the spleen, using β-actin as the loading control. (**B**) The ratio of p-NF-κB to NF-κB. (**C**–**E**) The relative expression levels of TNF-α, IL-1β, and IL-6 were normalized relative to β-actin. * *p* < 0.05, ** *p* < 0.01, **** *p* < 0.0001.

Figure 6 shows that protein expression levels of p-NF-κB/NF-κB, TNF-α, IL-1β, and IL-6 are significantly elevated in the CuD and CuD + CuSO_4_ groups compared to the control group. However, the CuD + CuSO_4_ group shows significantly lower expression levels of these proteins compared to the CuD group.

**Figure 7:**
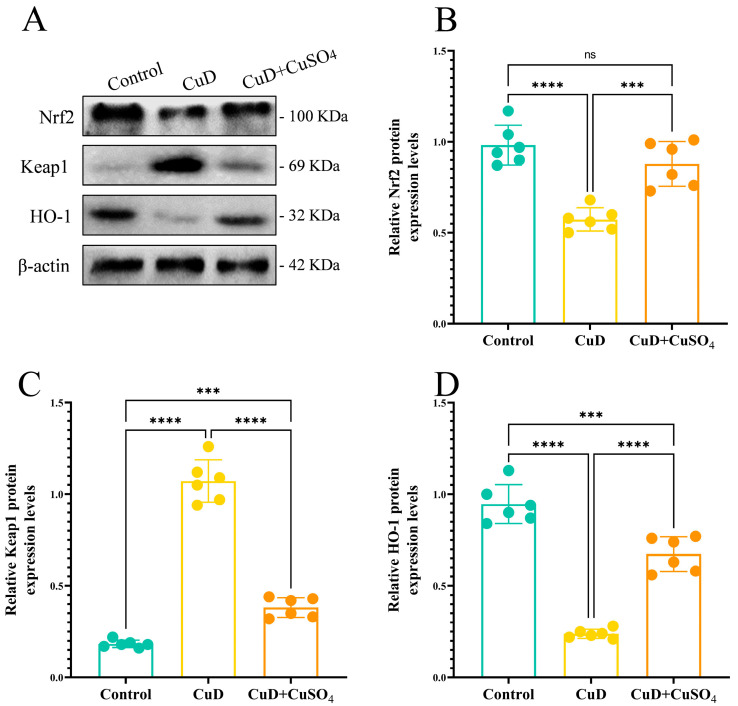
Effects of Cu deficiency and supplementation on protein expression of nrf2 signaling pathway of spleen. (**A**) Protein levels of Nrf2, Keap1, and HO-1 in spleen, using β-actin as loading control. (**B**–**D**) The relative expression levels of Nrf2, Keap1, and HO-1 were normalized to β-actin expression. *** *p* < 0.001, **** *p* < 0.0001, ^ns^ *p* > 0.05.

Figure 7 demonstrates that compared to the control group, the expression of Nrf2 and HO-1 proteins was significantly lower in the CuD group, while the expression of Keap1 was significantly higher. In the CuD + CuSO_4_ group, the expression of Nrf2 and HO-1 proteins was significantly increased, and the expression of Keap1 was significantly decreased compared to the CuD group. Additionally, the expression of Keap1 in the CuD + CuSO_4_ group was significantly higher than in the control group, while HO-1 expression was significantly lower, and Nrf2 expression showed no significant difference from the control group.

## 4. Discussion

Copper is an essential trace element vital for physiological processes like mitochondrial respiration, amino acid synthesis, and various biological reactions [3]. It serves as a structural component or cofactor for several oxidases to exert its antioxidant activity [23]. Additionally, copper acts as a modulator of immune cells, influencing immune function [24]. The spleen is one of the primary immune organs in the body. This study examines the impact of copper deficiency on mouse spleens, assesses the effects of subsequent copper supplementation, and explores the underlying mechanisms.

Food is the primary source of copper in the human body [25]. A copper-deficient diet was administered to mice to assess the effects of severe copper depletion on the spleen. The organ index serves as an essential phenotypic indicator of organ function. This study demonstrates that copper deficiency significantly reduces spleen volume and spleen index in mice, suggesting impaired spleen growth and development. The normal structure of an organ is closely related to its function, and structural damage often leads to functional changes. In this study, the structural changes observed in the spleen of copper-deficient mice, such as the reduction and blurring of white pulp, suggests that copper deficiency may impair the spleen’s normal architecture, which is essential for its immune functions. These findings align with earlier studies showing that copper deficiency impacts spleen lymphocyte differentiation and affects innate immune functions [26,27]. The results of this study show that, in the last week of copper supplementation, the spleens of CuD + CuSO_4_ mice showed less tissue damage and were closer to the control group, although there were no significant differences in the spleen index between the control, CuD, and CuD + CuSO_4_ groups. The intermediate spleen index in the CuD + CuSO_4_ group indicates that extra copper supplementation partially mitigated spleen damage from copper deficiency.

Copper is crucial for the body’s antioxidant system as it serves as a cofactor for enzymes like SOD. Therefore, copper deficiency may impair antioxidant functions. In this experiment, several typical markers of antioxidant function were measured. The findings indicated that copper deficiency in mice resulted in reduced T-AOC, SOD, and GSH levels, alongside elevated CAT and MDA levels in the spleen. Copper deficiency appears to lower antioxidant enzyme activity in the spleen, weaken antioxidant capacity, and impair the clearance of oxidative products, leading to increased oxidative product levels. Research has demonstrated that copper deficiency causes oxidative stress and liver damage in mice [20], and similar oxidative damage has been observed in the livers of rats [28]. Hawk et al. reported that under copper deficiency, the embryo malformation rate in copper-deficient rats was higher on day 12 of pregnancy, which could be attributed to oxidative damage, such as increased ROS levels and reduced SOD activity [29]. In this study, copper supplementation during the final week resulted in significant differences in CAT, SOD, and MDA levels between the CuD + CuSO_4_ group and the CuD group, indicating that copper supplementation alleviates oxidative damage in the spleen. While GSH and T-AOC levels did not show significant differences between the CuD + CuSO_4_ and CuD groups, there were some noted improvements. This could be due to the short supplementation period or insufficient copper sulfate injection dosage, which may not have shown a strong enough restorative effect.

Excessive ROS often leads to oxidative damage in the body and triggers the expression of inflammatory factors. These inflammatory factors promote the aggregation and activation of immune cells, enhancing the inflammatory response. On the other hand, during the inflammation process, immune cells produce large amounts of ROS, which helps clear pathogens and damaged cells but also exacerbates oxidative stress [30]. This study found that copper deficiency elevated the mRNA expression of key inflammatory enzymes, iNOS and COX2, along with the transcription factor NF-κB, which regulates the inflammatory response in mouse spleens. Copper deficiency significantly elevated IL-1β, IL-6, IL-8, and TNF-α levels in both peripheral blood and spleen, with increased mRNA and protein expression of these cytokines in the spleen, indicating a pronounced inflammatory response. The antioxidant system and inflammatory response in the body are closely interconnected. In this study, copper deficiency caused oxidative damage in the spleen, inhibited antioxidant enzyme activity, and increased the accumulation of oxidative products, which likely exacerbated oxidative stress and promoted the activation and expression of inflammatory molecules such as iNOS and COX-2. This subsequently triggered inflammatory cytokine production, potentially enhancing enzyme expression and establishing a positive feedback loop that exacerbated oxidative stress and inflammation. The hypothesis suggests that NF-κB may mediate oxidative stress and inflammation in the spleen of mice. NF-κB is an essential transcription factor that regulates immune and inflammatory responses by activating the expression of inflammation-related genes [31].

Nrf2 is a critical transcription factor primarily responsible for regulating cellular responses to oxidative stress. Nrf2 is typically inhibited by Keap1 binding; however, oxidative stress activates Nrf2, prompting its nuclear translocation and the transcription of antioxidant genes like HO-1, SOD, and GSH [32]. To explore the mechanisms underlying oxidative damage and inflammation in the spleen due to copper deficiency, the classic Nrf2/Keap1/HO-1 signaling pathway was investigated. The results showed that Nrf2 and HO-1 expression levels were significantly decreased in the spleens of copper-deficient mice, while Keap1 expression was increased. Previous research emphasizes the crucial role of trace elements like copper and zinc in the functioning of antioxidant enzymes and the regulation of redox signaling pathways, including Nrf2 [33]. This study found that copper deficiency markedly decreased Nrf2 and HO-1 expression and increased Keap1 expression in the spleen, suggesting that copper deficiency may weaken antioxidant defense by disrupting the Nrf2/Keap1/HO-1 pathway. This finding is consistent with Ngo and Duennwald’s review [34], which indicated that impairment of the Nrf2/Keap1 pathway may hinder antioxidant enzyme gene expression, reducing the body’s capacity to mitigate oxidative stress and leading to oxidative damage and cellular injury.

Copper, a crucial enzyme cofactor, is essential for activating Nrf2 and regulating the transcription of antioxidant genes. Some redox enzymes are regulated by Nrf2, and a decrease in Nrf2 expression may weaken the production of these antioxidants, making the body less able to cope with ROS accumulation. Copper deficiency significantly reduced antioxidant activity (e.g., SOD and GSH) in the spleen and increased MDA levels, indicating inhibition of the Nrf2 signaling pathway. This effect may be related to the inability of Nrf2 to effectively translocate to the nucleus or to its reduced ability to bind to antioxidant gene promoters.

Moreover, in this study, the expression of Keap1 was significantly elevated in copper-deficient mice. As a negative regulator of Nrf2, overexpression of Keap1 enhances its inhibitory effect on Nrf2, preventing its release and translocation to the nucleus to exert antioxidant regulatory functions. Studies indicate that Keap1 expression and stability under oxidative stress are linked to intracellular metal ion concentrations. Copper deficiency may lead to Keap1 accumulation by disrupting metal homeostasis, thus inhibiting Nrf2 pathway activation [35,36].

Copper deficiency may create a positive feedback loop of oxidative damage and inflammation, further inhibiting the Nrf2 pathway. Excessive ROS not only directly damages cell membranes and proteins but may also accelerate the accumulation of Keap1 or alter the stability of Nrf2 through oxidative modification. This study suggests that elevated inflammatory factors, including IL-1β and TNF-α, may enhance ROS production via downstream pathways such as NF-κB, thereby inhibiting the Nrf2 pathway.

## 5. Conclusions

Copper deficiency has a significant impact on the spleens of mice. The Nrf2/Keap1/HO-1 pathway is crucial in managing oxidative damage and inflammation caused by copper deficiency in the spleen. Copper supplementation can effectively mitigate the damage caused by copper deficiency by regulating this pathway. Therefore, maintaining appropriate copper levels in the body is crucial for regulating the activity of the Nrf2 pathway and protecting spleen function.

## Figures and Tables

**Table 1:** The primer sequences utilized in this study.

Gene	Forward (5′ → 3′)	Reverse (3′ → 5′)
IL-1β	TCGGCAAAGAAATCAAGATGGC	GTGCAAGTCTCATGAAGTGAGC
IL-6	ACAGAAGGAGTGGCTAAGGA	AGGCATAACGCACTAGGTTT
IL-8	CCTACTTCAGCATCCTCTACTGG	AGGGTTTCTTGAGAAGGGGAC
TNF-α	CGTCGTAGCAAACCACCAAG	TTGAAGAGAACCTGGGAGTAGACA
NF-κB	GGGCATGCGTTTCCGTTACA	ATGTGGATGAGGCCGGTGAG
iNOS	ATGGACCAGTATAAGGCAAGC	GCTCTGGATGAGCCTATATTG
COX2	TGCAGAATTGAAAGCCCTCT	CCCCAAAGATAGCATCTGGA
Nrf2	CTGAACTCCTGGACGGGACTA	CGGTGGGTCTCCGTAAATGG
Keap1	TCGAAGGCATCCACCCTAAG	CTCGAACCACGCTGTCAATCT
HO-1	AAGCCGAGAATGCTGAGTTCA	GCCGTGTAGATATGGTACAAGGA
β-Actin	GGCTGTATTCCCCTCCATCG	CCAGTTGGTAACAATGCCATGT

**Table 2:** Splenic histopathological damage scores of mice.

Histopathological Changes	Control	CuD	CuD + CuSO_4_
Decreased numbers in periarteriolar lymphoid sheath	0	4	2
Decreased numbers of splenic follicles	0	4	3
Decreased size of white pulp	0	3	2
Decreased size of marginal zone	0	3	2

The score ranges from 0 to 4, with 0 representing a normal organizational structure, and 1 to 4 representing minimal, mild, moderate, and marked changes, respectively.

## Data Availability

The article outlines the study’s original contributions, and the corresponding author can address further queries. The data are not publicly available due to privacy restrictions.
